# Anemia in Patients With Pituitary Adenomas: Prevalence and Correlation With Hypopituitarism

**DOI:** 10.7759/cureus.85422

**Published:** 2025-06-05

**Authors:** Diego Paixão Côrtes Aguiar, Alberto Chitolina Nesello, Bruna Araujo, Tainá Mafalda dos Santos, Vinicius Diniz Lima, Julia F Semmelmann Pereira Lima, Miriam da Costa Oliveira

**Affiliations:** 1 Neuroendocrinology Center, Santa Casa de Porto Alegre, Porto Alegre, BRA; 2 Graduate Program In Pathology, Federal University Of Health Sciences Of Porto Alegre (UFCSPA), Porto Alegre, BRA

**Keywords:** acromegaly, anemia, non-functioning pituitary adenoma, pituitary neoplasms, prolactinoma

## Abstract

Introduction: Reports on the prevalence of anemia in pituitary tumors are rare. This study aimed to establish the prevalence of anemia at the time of diagnosis in a cohort of patients with acromegaly, non-secretory pituitary adenomas (NFA), and prolactinomas. A secondary objective was to evaluate the coexistence of hypopituitarism and anemia.

Materials and Methods:* *Clinical and hematological data were reviewed in the medical records of patients diagnosed with pituitary adenomas at a neuroendocrinology reference center. Of the 344 cases eligible for the study, 98 (28.5%) were patients with acromegaly (mean age 50.5 years old, 57.1% women), 166 (48.3%) patients had NFA (64.6 years old, 44% women), and 80 (23.2%) had prolactinomas (52.1 years old, 60% women).

Results: Anemia was diagnosed in 68 (19.8%) patients: 22 (32.35%) with acromegaly (average hemoglobin (aHb): 11.8 g/dL), 29 (42.65%) with NFA (aHb: 11.6) and 17 (25%) with prolactinomas (aHb: 11.5). The prevalence was significantly higher in men, with 45 cases (27%), compared to 23 (13%) cases in women (p = 0.002). Most cases had mild anemia, 65 (96.6%). Regarding the classification according to mean corpuscular volume and mean corpuscular hemoglobin concentration values, 59 (87%) had normocytic normochromic anemia. Testosterone and thyroxine deficiency did not significantly affect the frequency of anemia. Anemia was associated with hypocortisolism (p= 0.027) and growth hormone deficiency (IGF-1) in the NFA group (p= 0.009).

Conclusion: This study shows a high prevalence of anemia, particularly in men, among patients with pituitary adenomas. The results improve knowledge of the comorbidities in patients with pituitary adenomas and suggest the importance of monitoring blood counts in patients’ evolution, given the impact of anemia on general health.

## Introduction

Anemia, a condition defined as a hemoglobin concentration below 13 g/dL in men and below 12 g/dL in women, affects approximately 24.3% of the global population [1]. Initial symptoms of anemia include fatigue and low resistance to exercise, which may worsen as the disease progresses.

Pituitary adenomas, especially macroadenomas, may develop with isolated or multiple hormonal deficiencies caused by tumor compression of adjacent tissues or secondary to surgical or radiotherapy treatment. The pituitary hormones are related to the hematopoietic system: hypothyroidism is linked to an increased prevalence of anemia, though the causality is still under debate [2,3]. The adult growth hormone (GH) deficiency is sometimes accompanied by normocytic normochromic anemia [4]; men who had hypogonadism tend to have lower hemoglobin values [5,6]; and mild anemia can be a warning sign of secondary adrenal insufficiency in patients with hypopituitarism [7]. However, reports about the relationship between anemia and pituitary adenomas are rare [8].

This study investigated the prevalence of anemia in patients with functioning and nonfunctioning pituitary adenomas and explored its association with hypopituitarism.

## Materials and methods

In this retrospective cross-sectional study, we reviewed clinical and hematological data at diagnosis in the medical records of patients diagnosed with pituitary adenomas at a Brazilian neuroendocrinology referral center. The sample was constructed using the inclusion criteria for diagnosing pituitary tumors based on clinical history, physical findings, imaging findings, and hormonal results. A second inclusion criterion was cases for whom it was possible to find hormonal data collected at the same time as the blood count prior to initiating hormone replacement to evaluate the coexistence of anemia and hypopituitarism. Exclusion criteria included the presence of pregnancy or acute or chronic infections, hematopoietic diseases, renal failure, malignant neoplasms, individuals who had undergone radiotherapy, and Cushing’s syndrome.

The collected variables were sex, age, tumor size, and serum levels of prolactin (PRL), thyroid-stimulating hormone (TSH), free thyroxin (FT4), adrenocorticotropic hormone (ACTH), cortisol, luteinizing hormone (LH), follicle-stimulating hormone (FSH), insulin-like growth factor 1 (IGF-1), and testosterone (men only). The assessed hematological parameters were the complete blood count test. Both hormone and hematological values were interpreted in conformity with the institutional laboratory reference values for morning collections, respecting the differences related to sex in the case of erythrocytes, hematocrit, hemoglobin, testosterone, and IGF-1, and age range for testosterone and IGF-1.

Acromegaly was diagnosed based on IGF-1 concentration above the normal ranges for age and gender, along with a lack of GH suppression after a glucose load. Prolactinoma was diagnosed when PRL levels exceeded 200 ng/mL in the presence of a pituitary lesion compatible with an adenoma. A nonfunctioning adenoma was defined when there was no evidence of hormonal hypersecretion. Hypopituitarism was diagnosed based on history and physical findings and hormonal results. Central hypothyroidism was indicated by low or inappropriately normal TSH, together with a low free T4. In the context of organic pituitary disease, IGF-1 levels less than the normal ranges for age and gender were considered sufficient for the diagnosis of GH deficiency without needing stimulation testing. Low morning cortisol level (<5 µg/dL) was classified as adrenal insufficiency. Hypogonadism in men was established when total testosterone was below the normal age-specific ranges. Hypogonadism in women was not evaluated due to insufficient clinical data regarding menstrual cycles and the use of sex steroids. According to the largest diameter, the tumor was characterized as microadenoma (<1 cm), macroadenoma (between 1 and 3.9 cm), and giant adenoma (≥ 4 cm).

Anemia was considered in the presence of hemoglobin below 13 g/dL in men and below 12 g/dL in women. The anemia was classified according to the mean corpuscular volume (MCV) and mean corpuscular hemoglobin concentration (MCHC) values [9], and its severity was determined using WHO criteria. According to it, mild anemia is when hemoglobin levels are between 12.9 and 11 g/dL in men and 11.9 and 11 g/dL in women; moderate between 10.9 and 8 g/dL in men and women; and severe below 8 g/dL in both [10].

The data study was performed using SPSS software version 25. Except for the variable age, in which the Student t-test was used, the analysis used the Chi-square test and Fisher’s exact test, considering significance p≤ 0.05. Continuous variables are presented as mean±SD.

The institution’s Ethics Committee approved the project under reference number 67925322.2.0000.5335, with written informed consent obtained.

## Results

In the 344 eligible cases for the study, the age ranged from 18 to 95 years, and 51.5% of the sample was female. Of the total, 98 (28.5%) were patients with acromegaly (mean age 50.5 years old, 57.1% women), 166 (48.3%) patients had NFA (mean age 64.6 years old, 44% women), and 80 (23.2%) had prolactinomas (mean age 52.1 years old, 60% women). Among patients with acromegaly, 73 (74.5%) had macroadenomas, and those with NFA or prolactinomas all had macroadenomas. Of the macroadenomas, 53 (16.6%) were giant adenomas, in 3 (3.1%) patients with acromegaly, 41 (24.7%) with NFA, and nine (11.3%) with prolactinomas.

Anemia was diagnosed in 68 cases (19.8%) of the total sample. Anemia was present in 29 (42.6%) patients with NFA, 17 (25%) of those with prolactinomas, and 22 (32.4%) patients with acromegaly, a similar percentage between groups (p= 0.627). Classifying by severity, 65 (95.6%) cases had mild anemia, and the remainder had moderate anemia.

The age did not differ in the anemic or non-anemic groups, respectively 56.9±16.7 and 57.8±15.6, p= 0.693. Among the patients with anemia, 45 (66.2%) were men, and 23 (33.8%) were women (p = 0.002). However, seven (77.8%) patients with moderate anemia were women. No difference was observed in the presence of anemia between patients with giant macroadenomas and other macroadenomas, nor in NFA, where six (14.7%) patients who had giant adenomas were anemic, nor in prolactinomas, where one (11.1%) had anemia (respectively, p=0.753 and p=1.00). Also, no divergence was observed between the hemoglobin average level among the giant adenomas (13.5±1.2) and the other macroadenomas (13.3±1.3) (p= 0.209).

The anemia was classified as normocytic normochromic in 59 (86.7%) cases and as microcytic normochromic in six (8.8%). It was normocytic hypochromic in one case (1.5%), macrocytic normochromic in another (1.5%), and microcytic hyperchromic in a third case (1.5%) (Table 1). The classification of anemia did not differ among the various tumor types (p = 0.505).

**Table 1:** Anemia classification in pituitary tumors NFA: nonfunctioning adenoma; PRL: prolactinoma

Anemia Type	Acromegaly	NFA	PRL	Total
	n (%)	n (%)	n (%)	n (%)
Normocytic, normochromic	20 (90.9)	25 (86.2)	14 (80)	59 (86.7)
Normocytic, hypochromic	-	1 (3.4)	-	1 (1.5)
Microcytic, normochromic	1 (4.5)	2 (6.9)	3 (20)	6 (8.8)
Macrocytic, normochromic	-	1 (3.4)	-	1 (1.5)
Microcytic, hyperchromic	1 (4.5)	-	-	1 (1.5)
All subtypes	22 (32.4)	29 (42.6)	17 (25)	68 (100)

Table 2 shows the number and percentage of patients with hormonal deficiencies according to adenoma type in the patients with available hormonal levels. The prevalence of hypogonadism did not differ among the different hormonal tumor types (p = 0.742). GH deficiency was more frequent in NFAs than in prolactin-secreting tumors (p = 0.007). Cortisol deficiency and hypothyroidism were more prevalent in patients with NFA compared to those with acromegaly or prolactinoma.

**Table 2:** Prevalence of hormonal deficiencies in the study groups *Number of patients evaluated **Chi-square test NFA: nonfunctioning adenoma; PRL: prolactinoma; NA: not applicable

Hormone Deficiency	Acromegaly	NFA	PRL	Total	P value**
	n (%)	n (%)	n (%)	n (%)	n
Testosterone (163)*	26 (72.2)	60 (72.3)	23 (79.3)	109 (73.6)	0.742
GH (242)*	NA	52 (49.1)	10 (23.3)	62 (41.6)	0.007
Cortisol (298)*	11 (12.6)	38 (26)	9 (13.8)	58 (19.5)	0.019
Thyroxine (317)*	3 (3.4)	24 (15)	5 (7.4)	32 (10.1)	0.010

Testosterone was low in 109 (73.6%) of the men. Anemia was observed in 31 (28.4%) patients with low testosterone and in seven (18%) with normal testosterone levels, with no difference (p= 0.278).

Low IGF-1 was detected in 52 cases (49.1%) of NFA and 10 cases (23.3%) of prolactinomas. In NFA with low IGF-1, 15 (28.8%) had anemia, while in the remainder of the group, the frequency of anemia was significantly lower, with 4 (7.4%) cases. (p=0.009). In prolactinomas, anemia did not diverge between patients who had or did not have low IGF-1 (p=0.127).

Low cortisol was present in 58 (19.5%) cases. Anemia was observed in 18 (31%) patients with cortisol deficit and 41 (17%) with normal cortisol. There was a significantly higher prevalence in the hypocortisolemic group (p = 0.027).

FT4 levels were low in 32 (10.1%) of the sample. The occurrence of anemia did not differ according to the presence or absence of hypothyroidism (p=0.292).

Therefore, anemia was significantly associated with glucocorticoid deficiency and, in NFAs, with GH deficiency, with no association observed with other hormonal deficiencies.

## Discussion

Few studies clearly show the worldwide prevalence of anemia, stratifying by gender and age, making the comparison with the population of the present research difficult. Global data show that anemia affects approximately 27% of the population [11]; when considering all ages and genders, the prevalence is around 24.3% [1]. In Brazil, considering only the age group between 15 and 59 years old, 10.5% of men are affected by the condition, while in women, this number reaches 22.6% [12]. A recent national study indicates that the prevalence in the adult and elderly population (excluding pregnant women) is 9.9% (7.2% in men and 12.3% in women) [13]. In our study, compared with the national estimate, we found that the prevalence of anemia is three times higher in men with pituitary adenomas. Although iron deficiency is the dominant cause of anemia (60%) in the general population, other vital causes include hemoglobinopathies, infections, chronic kidney diseases, and gastrointestinal and gynecologic conditions [11]. In our study, the two most prevalent types of anemia are those generally related to chronic diseases.

Rare studies have evaluated the presence of anemia in pituitary tumors, limited primarily to its association with hypogonadism. A significantly distinct prevalence of anemia was reported in men (84%) and women (16%) in a group of 216 patients with pituitary adenomas [8]. This finding was confirmed in the current study. However, for these authors, 46.3% of men with low testosterone were anemic, a percentage considerably different from the others, a divergence that was not found in our series.

Some isolated reports of anemia associated with prolactinomas have been described by MacLean and Hanley [14] in a 70-year-old man who had chronic anemia initially ascribed to old age and by Iglesias and Díez [15] in a 58-year-old man with macroprolactinoma and hypopituitarism. In both cases, the anemia disappeared after the resolution of hyperprolactinemia or testosterone replacement.

Two small series of prolactinomas have been described, in which the presence of anemia was related to hypogonadism/hypopituitarism. In assessing 26 men with prolactinomas, lower hemoglobin concentrations were observed in those with macroadenomas [16]. Anemia was present in 34.6% of the cases, all associated with macroprolactinomas. Hemoglobin concentration was significantly lower in hypogonadal men, and anemia was related to hypopituitarism. Mild normocytic normochromic anemia is found in about a third of men with prolactinomas, according to the authors [16]. In another series of 36 men with hyperprolactinemia, both the mean testosterone level and the mean hemoglobin level were low, in four cases equal to or less than 11.5 g/dL, and hemoglobin increased after prolactin suppression with cabergoline [5].

According to Duskin-Bitan, mild anemia is common in men with macroprolactinomas. It is associated with hypogonadism and usually improves following successful treatment and normalization of prolactin and testosterone. On the other hand, patients with other pituitary adenomas may also have hyperprolactinemia due to the interruption of the pituitary stalk [17].

In a cohort of 70 males with prolactinomas and hypogonadism, hemoglobin levels started to decline a median of 6.1 years before the diagnosis of prolactinoma. On average, there was a 4.1-year gap between the drop in hemoglobin levels and the onset of hypogonadal symptoms. These results suggest that hemoglobin decline before prolactinoma diagnosis may serve as a marker for hyperprolactinemia onset, allowing for a more accurate evaluation of disease duration [18]

There is a report in which the presence of anemia does not correlate with tumor size in women, while in men, it predominates in those who have macroadenomas [8]. On the other hand, in the Shimon and colleagues’ observation, baseline hemoglobin levels inversely correlated with tumor size, reaching levels of 13.7 g/dL in 10 men with macroprolactinomas of 10-20mm in size, 13.0 g/dL in 18 subjects with macroadenomas of 21-40mm and 12.4 g/dL in seven patients with giant prolactinomas (>40 mm) [5]. Here, there was no difference in the level of hemoglobin and the presence of anemia according to the tumor size (considered macroadenomas and giant adenomas).

Considering the possible consequences of a deficiency of pituitary hormones, mild anemia in hypopituitarism could also be expected as part of secondary adrenal insufficiency and hypothyroidism [7]. While the association with secondary adrenal insufficiency has been confirmed, the association with hypothyroidism was not observed in our series. The increase in cases of anemia was also not observed in hypogonadism, in which the androgens stimulate erythropoiesis and raise levels of red cells, hemoglobin, and hematocrit [19]. Notably, the GH deficiency was related to anemia only in the NFA group.

The limitations of the present study include its retrospective design and the lack of consideration for comorbidities that could be associated with anemia, beyond those included in the exclusion criteria. Additionally, iron, vitamin B12, and folate levels were not measured. Another partial limitation is the absence of hematimetric reassessment following the replacement of deficient pituitary hormones. Reassessing hematimetric parameters would allow for evaluating the effect of levothyroxine and glucocorticoid replacement on anemia in these cases.

## Conclusions

In conclusion, our findings indicate that anemia is more prevalent in men with pituitary adenomas and appears to be particularly associated with hypocortisolism. These results enhance our understanding of the epidemiological profile of pituitary adenoma patients in terms of hematimetric parameters. Considering the impact of anemia on overall health and the possibility of it representing another comorbidity associated with pituitary tumors, it is necessary to confirm the current findings in new and expanded groups.
